# Solitary circumscribed neuroma of the conjunctiva: Differential diagnosis from neurofibroma is a must?

**DOI:** 10.5935/0004-2749.20200084

**Published:** 2024-02-11

**Authors:** Pelin Kiyat, Ozlem Barut Selver, Taner Akalin, Melis Palamar

**Affiliations:** 1 Department of Ophthalmology, Ege University, Izmir, Turkey; 2 Department of Pathology, Ege University, Izmir, Turkey

**Keywords:** Neuroma, Multiple endocrine neoplasia type 2b, Conjunctival neoplasms, Diagnosis, differential, Human, Case reports, Neuroma, Neoplasia endócrina múltipla tipo 2b, Neoplasias da túnica conjuntiva, Diagnostico diferencial, Humanos, Relatos de casos

## Abstract

A 42-year-old male presented with a 4-week history of a mass in the right inferior palpebral conjunctiva close to the punctum. An excisional biopsy of the lesion and histopathological examination revealed that the mass was composed of Schwann cells with thin conical nuclei, fine chromatin, and unnoticeable nucleoli. Immunohistochemically, the spindle cells were diffusely and strongly positive for S100 protein. Neurofilament immunostaining was also positive, which highlighted axons. In light of these findings, the tumor was diagnosed as solitary circumscribed neuroma. A comprehensive evaluation for multiple endocrine neoplasia type 2b was performed. However, no multiple endocrine neoplasia type 2b stigmata and no family history were detected. The diagnosis was therefore finalized as solitary circumscribed neuroma, which is considered as a rare condition. The differential diagnosis is based on the histopathological examination and immunohistochemical evaluation. As the tumor can be related with multiple endocrine neoplasia type 2b, it is essential to systematically investigate for multiple endocrine neoplasia type 2b in such cases.

## Abstract (pt)

Um homem de 42 anos apresentou uma massa na conjuntiva palpebral inferior direita, próxima ao punctum, com evolução de 4 semanas. Uma biópsia excisional da lesão e o subsequente exame anatomopatológico revelaram que a massa era composta de células de Schwann com núcleos cônicos, croma tina fina e nucléolos não visíveis. Ao exame imuno-histoquímico, as células fusiformes mostraram-se difusa e fortemente positivas para a proteína S100. A imunocoloração também foi positiva para neurofilamentos e evidenciou os axônios. Considerando esses achados, o tumor foi diagnosticado como um neuroma circunscrito solitário. Procedeu-se uma investigação completa para neoplasia endócrina múltipla tipo 2b, entretanto, estigmas característicos e história familiar não foram detectados. Assim, o diagnóstico foi firmado como neuroma circunscrito solitário, condição rara cujo diagnóstico diferencial baseia-se no exame anatomopatológico e na avaliação imuno-histoquímica. Já que esse tumor pode estar relacionado à neoplasia endócrina múltipla tipo 2b, torna-se essencial, nesses casos, a investigação da neoplasia de forma sistemática.

## INTRODUCTION

Schwann cells and endoneural and perineural fibroblasts are the main components of peripheral nerve sheath cells^(1)^. Neurofibromas, schwannomas, and neuromas are the types of benign peripheral nerve sheath tumors. The composition of the proliferating cells defines the type of the tumor^(2)^. All these three tumors present as round-shaped, well-defined lesions where clinical differentiation is impossible. Immunohistochemical staining aids in classifying the type of tumor.

Neuromas are benign peripheral nerve sheath tumors characterized by a combined proliferation of Schwann cells, perineural fibroblasts, and axons^(3)^. The tumors are usually located in the facial skin and detected in the third or fourth decades of life^(4)^. Although ocular involvement is quite rare in solitary circumscribed neuromas, if they occur, they are located on the eyelid or conjunctiva. The tumor is usually encapsulated, but not always^(5)^.

With this study, we hereby aim to report a case with a solitary circumscribed conjunctival mass, pathologically diagnosed as neuroma, and to discuss the clinical and histopathological characteristics related to the case.

## CASE REPORT

**Figure 1:**
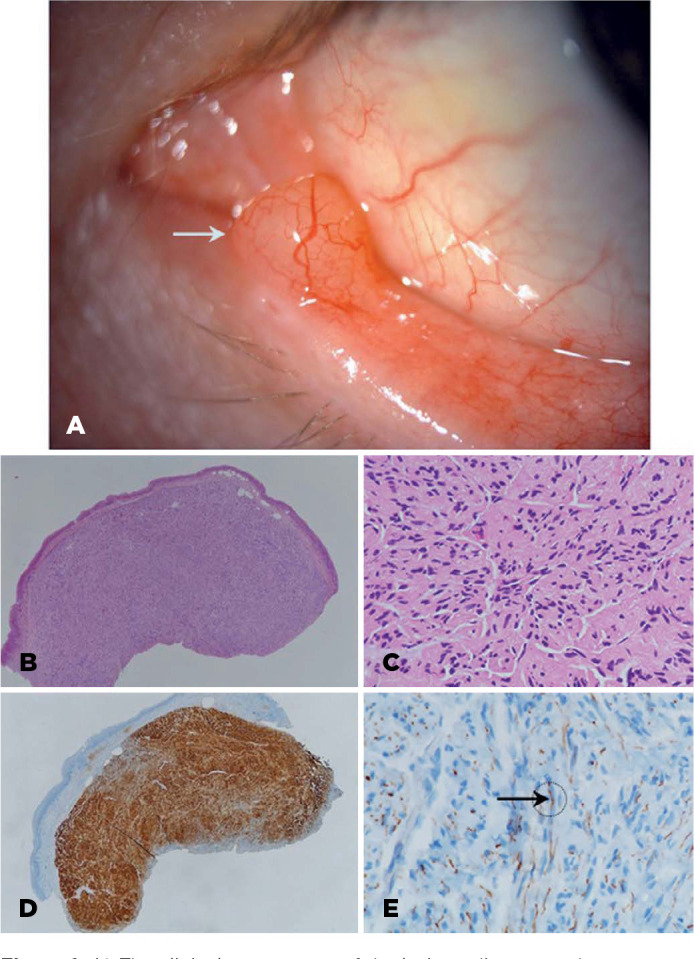
A) The clinical appearance of the lesion adjacent to the punctum: dome-shaped, elevated, amelanotic palpebral conjunctival lesion with a smooth surface. B) At scanning magnification, tumor was characterized with well-circumscribed nodule. C) Nodule was composed of Schwann cells with slender tapered nuclei, fine chromatin, and inconspicuous nucleoli. Spindle cells were arranged in intersecting fascicles that are separated by clefts focally. D) Immunohistochemical evaluation. The spindle cells were diffusely and strongly positive for S100 protein, which is positive for neural-based cells. E) Immunohistochemical evaluation. A neurofilament immunostaining highlighting a number of axons within fascicles

A 42-year-old male presented with a 4-week history of a painless mass in the right inferior palpebral conjunctiva close to the punctum (Figure 1A). No history for systemic disease and trauma associated with the orbita existed.

Through a slit-lamp examination, a round-shaped, elevated, amelanotic palpebral conjunctival lesion with a smooth surface near the punctum was observed. There was no sign of irritation such as the presence of chemosis or feeder vessels.

A total excisional biopsy of the lesion under local anesthesia was performed. On gross examination, the lesion appeared as a pink mass and measured 0.5 × 0.3 × 0.3 cm. The histopathological examination revealed that it was composed of Schwann cells with thin conical nuclei, fine chromatin, and unnoticeable nucleoli. (Figure 1B). Spindle cells were organized in crossed design which are divided into sections by focal clefts (Figure 1C). Immunohistochemically, the spindle cells were positive for S100 protein diffusely and strongly (Figure 1D). Neurofilament immunostaining was also positive, which highlighted a number of axons within fascicles (Figure 1E). In light of this, solitary circumscribed neuroma was diagnosed.

Therefore, a comprehensive evaluation for MEN-2b, which involved an endocrinologist, gastroenterologist, and orthopedist, was performed. As no MEN-2b stigmata was detected and no family history of MEN-2b was present, the mass was proven to be a solitary circumscribed neuroma of the conjunctiva.

At the 12-month follow-up visit, the patient remained healthy with no recurrence or evidence of MEN-2b.

## DISCUSSION

The solitary circumscribed neuroma is a non-hereditary proliferation of Schwann cells and perineural fibroblasts where MEN-2b is absent. The tumor appears in the third or fourth decade of life, and it is usually encapsulated where a diagnosis is only possible via a histopathological examination^(6)^. Solitary circumscribed neuroma of the conjunctiva can be managed with a

total excisional biopsy, which allows for histopathologic evaluation and prevents recurrences. After the surgery, there was no recurrence in our case; however an incomplete biopsy can result in recurrences.

MEN-2b is a rare syndrome, which can be associated with conjunctival neuromas. For this reason, when a neuroma of the conjunctiva is detected, it should prompt the clinician to analyze the patient for MEN-2b. The consultations should include endocrinologic, gastroenterologic, and orthopedic examinations. This syndrome which was first described 90 years ago, together with multiple mucosal neuromas, medullary thyroid carcinoma, pheochromocytoma and marfanoid body features. Mucosal neuromas are the earliest sign, and they occur in most patients. Neuromas generally appear on the lips, tongue, and buccal mucosa. Eyelids and conjunctiva rarely develop neuromas. Gastrointestinal ganglioneuromatosis is common and affects the gastrointestinal motility, which can result in either constipation or diarrhea. Medullary thyroid carcinoma tends to be aggressive in MEN-2b cases and usually results in the death of these patients^(7)^.

Clinical differential diagnosis of conjunctival neuroma is not possible. Non-neural tumors - such as cha lazion and epidermoid cyst - which appear as subconjunctival light yellow cysts should be considered for differential diagnosis. In a patient with recurrent chalazion, meibomian gland adenocarcinoma should be considered first; thereafter peripheral nerve sheath tumors should be considered as well. Chalazion includes inflammatory lipogranulation tissue, and keratin is the main component in the epidermoid cyst^(8)^. Our case is a solitary circumscribed neuroma, which contains Schwann cells and perineural fibroblasts. Poonam and associates^(9)^ presented a solitary neurofibroma of the eyelid, mimicking a tarsal cyst. In that case, the mass was on the upper eyelid, so the lesion appearance was resembling a chalazion. In the presented case, the lesion was located adjacent to the punctum, which is not a very common location for a chalazion. The differential diagnosis also includes epibulbar dermoid, leiomyoma, fibrous histiocytoma, lymphoma, myxoma, and other benign peripheral nerve sheath tumors, all of which can only be discriminated with histopathological examination^(10)^.

Benign peripheral nerve sheath tumor differentiation is only possible with immunohistochemistry. Ishida and associates^(11)^ described a solitary circumscribed neu roma of the conjunctiva. The histopathological and immunohistochemical evaluations were very close to the presented case’s findings, including positive staining for S100, a specific marker for neural-based cells; for neurofilament which shows axonal filaments; and for epithelial membrane antigen (EMA) which shows the capsule. Conversely, neurofibromas are non-encapsulated lesions with no staining for EMA. Few cells show positive staining for S100. Schwannomas are often encapsulated tumors, which show positive staining for S100 and negative staining for neurofilament. In light of these immunohistochemical findings, our patient was diagnosed of neuroma. Dubovy et al.^(12)^ described solitary circumscribed neuroma of the eyelid in two cases. Although their cases were older with different lesion locations, eventually the histopathological and immunohistochemical results were similar, and there was no recurrence. It is especially important to differentiate a neuroma from other peripheral nerve sheath tumors because neuromas are benign tumors without the risk of malignant transformation. However, deeply located, large neurofibromas associated with neurofibromatosis (NF) have malignant transformation risk. In the presented case, there was no evidence for pleomorphism or mitotic activity, which are the main markers for malignancy. Neuromas can be associated with MEN-2b and neurofibromas - especially the plexiform type - and can be related to NF. For this reason, the discrimination between these two tumors which depends on the histopathologic evaluation will change the follow-up of the patient.

In conclusion, solitary circumscribed neuroma is quite a rare condition. The differential diagnosis is based on the histopathological examination and immunohistochemical evaluation. As these tumors can be related to MEN-2b, systemic examination for MEN-2b in these patients is essential.
